# Nigrosporins B, a Potential Anti-Cervical Cancer Agent, Induces Apoptosis and Protective Autophagy in Human Cervical Cancer Ca Ski Cells Mediated by PI3K/AKT/mTOR Signaling Pathway

**DOI:** 10.3390/molecules27082431

**Published:** 2022-04-09

**Authors:** Jing Zhang, Zhi-Yong Guo, Chang-Lun Shao, Xue-Qing Zhang, Fan Cheng, Kun Zou, Jian-Feng Chen

**Affiliations:** 1 Hubei Key Laboratory of Natural Products Research and Development, College of Biological and Pharmaceutical Sciences, China Three Gorges University, Yichang 443002, China; zj031020@163.com (J.Z.); zhyguoctgu@foxmail.com (Z.-Y.G.); happy.xueqing@163.com (X.-Q.Z.); fancy1351@163.com (F.C.); kzou@ctgu.edu.cn (K.Z.); 2 Key Laboratory of Marine Drugs, School of Medicine and Pharmacy, Ocean University of China, Ministry of Education of China, Qingdao 266003, China; shaochanglun@163.com

**Keywords:** Nigrosporins B, apoptosis, autophagy, PI3K/AKT/mTOR, cervical cancer

## Abstract

Nigrosporins B, an anthraquinone derivative obtained from the secondary metabolites of marine fungus *Nigrospora oryzae*. In this study, we characterized the distinctive anti-cancer potential of Nigrosporins B in vitro and underlying molecular mechanisms in human cervical cancer Ca Ski cells for the first time. The results of MTT assay showed that Nigrosporins B significantly inhibited the proliferation of multiple tumor cells in a dose-dependent manner, especially for the Ca Ski cells with an IC_50_ of 1.24 µM. Nigrosporins B exerted an apoptosis induction effect on Ca Ski cells as confirmed by flow cytometry, AO/EB dual fluorescence staining, mitochondrial membrane potential analysis and western blot assay. In addition, Nigrosporins B induced obvious autophagy accompanied with the increase of autophagic vacuoles and the acceleration of autophagic flux as indicated by Cyto-ID staining, mRFP-GFP-LC3 adenovirus transfection and western blot analysis. Interestingly, the combination of Nigrosporins B with the three autophagy inhibitors all significantly enhanced the cytotoxicity of Nigrosporins B on Ca Ski cells, indicating that the autophagy induced by Nigrosporins B might protect Ca Ski cells from death. Furthermore, we found that Nigrosporins B inhibited the phosphorylation of PI3K, AKT, mTOR molecules and increased the protein expression levels of PTEN and p-AMPKα in a dose-dependent manner, suggesting that Nigrosporins B induced apoptosis and protective autophagy through the suppression of the PI3K/AKT/mTOR signaling pathway. Together, these findings revealed the anti-cervical cancer effect of Nigrosporins B and the underlying mechanism of action in Ca Ski cells, it might be as a promising alternative therapeutic agent for human cervical cancer.

## 1. Introduction

Cervical cancer is one of the most common malignancies in women worldwide [1]. The current clinical treatment is multifaceted and may include a combination of surgery, radiotherapy, chemotherapy and human papillomavirus (HPV) vaccines [2], but efficacious treatments for this pathological condition are still challenging. In particular, the drug resistance and serious side effects of chemotherapy often lead to poor treatment expectations [3]. Thus, further exploration of novel effective and less toxic chemotherapeutic agents for the treatment of cervical cancer is still very necessary.

Most of chemotherapy drugs primarily act by triggering the programmed cell death (PCD) pathways to kill tumor cells directly [4]. Targeting the process of PCD with smallmolecule compounds has became a promising cancer therapeutic strategy over the last decades [5]. Apoptosis and autophagy, as main two forms of PCD, play significant physiological roles in cellular survival, stress adaptation and the development of tumor [6]. Apoptosis is well known as a key tumor suppressor mechanism and most anticancer drugs currently used in clinical mainly exploit the intact apoptotic signaling pathways to trigger cancer cell death [7]. However, different from apoptosis, it is worth noting that autophagy is generally considered a “double-edged sword” in the context of cancer progression: autophagy activation can promote cancer cells survival (protective autophagy), or contribute to cancer cell death (cytotoxic/nonprotective autophagy) [8]. So, understanding the molecular mechanisms that drug-induced PCD in tumor cells may provide novel opportunities for anti-cancer drugs development.

Although there were obvious distinction in morphological characteristics and biochemical mechanisms, the close relationship and crosstalk between apoptosis and autophagy have been widely reported [9]. For example, they regulated by the common upstream signaling pathways or regulatory elements, such as PI3K/AKT/mTOR pathway [10], AMPK [11] and p53 protein [12], and Bcl-2-Beclin 1 complex molecules [13], et al. Among this, PI3K/AKT/mTOR signaling pathway received more attention owing to it involves in a broad range of cellular processes such as survival, proliferation, growth, metabolism, angiogenesis and metastasis [14]. In addition, PI3K/AKT/mTOR signaling pathway is one of the most frequently activated pathway in various human cancers and plays a crucial role in promoting tumor initiation and progression [15]. Targeting PI3K/AKT/mTOR-mediated cell death has became an important therapeutic strategy in enhancing the chemo-sensitivity and avoiding drug resistance for multiple tumors [16].

**Figure 1:**
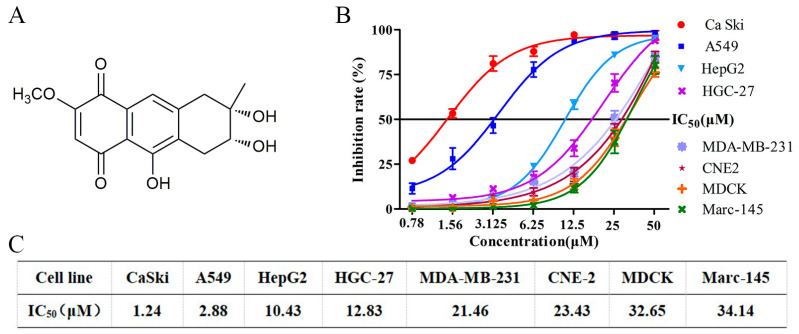
(**A**) The chemical structure of Nigrosporins B; (**B**) The Cytotoxicity of Nigrosporins B on Ca Ski, A549, HepG2, HGC-27, MDA-MB-231, CNE2, MDCK and Marc-145, the cells were treated with Nigrosporins B of different concentrations (50, 25, 12.5, 6.25, 3.125, 1.56, 0.78 µM) for 48 h, and cell viability was measured by MTT assay; (**C**) The IC_50_ of Nigrosporins B on Ca Ski, A549, HepG2, HGC-27, MDA-MB-231, CNE2, MDCK, Marc-145.

In recent years, the marine natural products have attached more attention due to their novel structures and significant biological activities [17], especially for the secondary metabolites of marine-derived fungus, which has became an important source of innovative anti-cancer drug development and numerous candidate drugs with multi-targets and low toxicity have been discovered [18]. Nigrosporins B (Figure 1A), an naphthoquinone derivative, was isolated by our research group from a sponge-associated fungus *Nigrospora* sp. that derived from an unidentified sea anemone in Yongxing island of Xisha sea area [19]. Previous studies have shown that Nigrosporins B possesses the certain phytotoxic activities [20] and cytotoxic activities [21], et al. However, there is not any report on its anti-cervical cancer activity and underlying mechanism.

Based on this, this study would aim to assess the anti-tumor activity of Nigrosporins B for various human tumor cells and focus on the process of PCD characterized by apoptosis and autophagy and the signaling pathway of PI3K/AKT/mTOR to deeply clarify the molecular mechanisms. Besides, the further efforts have also been carried out to explore the effective measures for improving the anti-cervical cancer potential of Nigrosporins B. In brief, we investigated the relevant mechanism of Nigrosporins B in inhibiting the proliferation of cervical cancer cells, these will help for the future clinical application of Nigrosporins B.

## 2. Material and Method

### 2.1. Cell Lines and Cultures

Human cervical cancer Ca Ski cells, human lung cancer A549 cells, human hepatocellular carcinoma HepG2 cell, human gastric carcinoma HGC-27 cells, human mammary carcinoma MDA-MB-231 cells, human nasopharyngeal carcinoma CNE-2 cells, African green monkey kidney Marc-145 cells, Madin-Darby canine kidney MDCK cells were purchased from the cell bank of the Shanghai Institute for Biological Sciences, Chinese Academy of Sciences (Shanghai, China) and maintained in RPMI-1640, L-15 or DMEM culture medium supplemented with 10% fetal bovine serum at 37 °C with 5% CO_2_ in a humidified incubator.

### 2.2. Chemicals and Reagents

Reagents for cell culture were obtained from Gibco BRL Life Technologies (Grand Island, NY, USA). 3-(4,5-dimethylthiazolyl-2)-2,5-diphenyltetrazolium bromide (MTT) were purchased from Sigma chemical (St. Louis, MO, USA). The autophagy inhibitors 3-methyladenine (3-MA), chloroquine (CQ) and bafilomycin A1 (Baf A1) were purchased from Medchem Express Co., Ltd. (Monmouth Junction, NJ, USA). Rabbit monoclonal antibodies against Bax, Bad, Bcl-2, Bcl-xL, Caspase-3/7/9, Cleaved Caspase-3/7/9, LC3-I/II, Beclin 1, p-Beclin 1, ULK1, p-ULK1, p62, ATG 3, ATG 4B, ATG 5, ATG 7, ATG 12, PI3K, p-PI3K, AKT, p-AKT, mTOR, p-mTOR, AMPKα, p-AMPKα, PTEN, β-actin and HRP-conjugated secondary antibody were purchased from Cell Signaling Technology (Dancer, MA, USA).

### 2.3. MTT Assay

Cells in the exponential phase were seeded in 96-well microplates at 1 × 10^5^ cells/well and treated with two-fold dilution concentrations of Nigrosporins B from 50 µM to 0.78 µM for 48 h before the MTT assay. Cytotoxicity was expressed as the 50% inhibition concentration (IC_50_).

### 2.4. Flow Cytometry Assay (FCM)

An Annexin V-fluorescein isothiocyanate (FITC)/propidium iodide (PI) apoptosis detection kit (Enzo Life Sciences, Farmingdale, NJ, USA) was used to quantitative assess the percentage of apoptotic cells by flow cytometry. After treated with Nigrosporins B of different time and concentration, Ca Ski cells were gathered and washed twice with cold PBS, and then stained with Annexin V-FITC/PI at room temperature in the dark for 15 min and analyzed by a BD FACSCalibur flow cytometer (BD Biosciences, Franklin Lakes, NJ, USA).

### 2.5. Acridine Orange/Ethidium Bromide (AO/EB) Dual Fluorescence Staining

Apoptosis cells were qualitative evaluated by an AO/EB dual fluorescence staining kit (Beyotime Biotechnology, Shanghai, China). Briefly, the Ca Ski cells were seeded in 24-well microplates at 5 × 10^5^ cells/well and treated with Nigrosporins B of different concentration for 24 h, the cells were washed twice with dyeing buffer and then stained with AO and EB dye in the dark at 37 °C for 20 min. Images were taken by a fluorescence microscope (Olympus, Tokyo, Japan) and analyzed by Image-Pro-Plus version 7.0 software (Media Cybernetics, Rockville, MA, USA).

### 2.6. Mitochondrial Membrane Potential (MMP) Assay

JC-1 fluorescence mitochondrial imaging was used to assess the depolarization of MMP in Ca Ski cells. According to the method provided by the manufacturer (Beyotime Biotechnology, Shanghai, China), Ca Ski cells were treated with Nigrosporins B and incubated with JC-1 dye solution for 20 min at room temperature, and then washed twice with dyeing buffer. Images were taken by a fluorescence microscope (Olympus, Tokyo, Japan).

### 2.7. Autophagic Vacuoles Detection

A Cyto-ID autophagy detection kit (Enzo Life Sciences, Farmingdale, NJ, USA) was used to detect the presence of autophagic vacuoles in live cells by applying a dye that can excite brightly fluorescent in vesicles that formed in the process of autophagy activation, which has been proved to be a reliable method to monitor the early stages of autophagy activation [22]. According to the method provided by the manufacturer, the Ca Ski cells were treated with Nigrosporins B for 24 h, collected and washed twice with cell culture medium, then resuspended in assay buffer and added the diluted Cyto-ID dye solution, incubated for 30 min at room temperature in the dark. The autophagic vacuoles were analyzed by flow cytometry (BD Biosciences, Franklin Lakes, NJ, USA) in the FL1 channel.

### 2.8. Autophagic Flux Assessment

To assess autophagic flux in response to Nigrosporins B activation, a tandem mRFP-GFP-LC3 adenovirus (Hanheng Biotechnology Co Ltd., Shanghai, China) was transfected into Ca Ski cells for 6.5 h at a MOI of 200 before treated with Nigrosporins B for 18 h. Then, cells were observed under a laser confocal fluorescence microscope (LCFM) (Olympus FV1200, Tokyo, Japan). Autophagic cells with more than five RFP-GFP-LC3 dots were recorded and the images were takened.

### 2.9. Western Blot Analysis

Ca Ski cells were treated with Nigrosporins B of 0, 2.5, 5 and 10 µM for 24 h. Total protein were extracted, quantified and loaded on a 10% SDS-gel. Subsequently, proteins were transferred to PVDF membranes. After blocking, membranes were incubated with the primary antibodies at 4 °C overnight, washed five times and incubated with the secondary antibody for 1.5 h at 37 °C. Signals were visualized by ECL (Beyotime Biotechnology, Shanghai, China), and produced by a Tanon 5200 luminescence imaging system (Tanon Technology Co., Ltd., Xinjiang, China). Densitometry analysis was performed by ImageJ version 2.1 (National Institutes of Health) and using β-actin as the control load.

### 2.10. Statistical Analysis

All data are presented as the mean ± standard deviation of three independent experiments. Statistical analysis was performed by a one-way ANOVA with GraphPad Prism 6.0 software (GraphPad Software, Inc., La Jolla, CA, USA). *p* < 0.05 was considered to indicate a statistically significant difference.

## 3. Results

### 3.1. Nigrosporins B Inhibited the Proliferation of Ca Ski Cells In Vitro

To evaluate the anti-tumor potential of Nigrosporins B, various human tumor cells and normal cells were exposed to 0.78–50 µM of Nigrosporins B for 48 h, and cell viability was examined by MTT assay. As shown in Figure 1B, Nigrosporins B exhibited a broad spectrum of inhibition on the tested tumor cells in a dose-dependent manner. Notably, the human cervical cancer Ca Ski cells were more sensitive to Nigrosporins B with an IC_50_ value of 1.24 μM, and followed by A549, HepG2, HGC-27, MDA-MB-231 and CNE-2 cells with the IC_50_ values of 2.88, 10.43, 12.83, 21.46 and 23.43 μM, respectively (Figure 1C). Moreover, two types of normal cells (MDCK and Marc-145) also been examined and Nigrosporins B exhibited less toxicity towards them with the IC_50_ value of 32.65 and 34.14 μM (Figure 1C). Accordingly, the Ca Ski cells were used in subsequent experiments as a representative to research the anti-tumor mechanism of Nigrosporins B.

### 3.2. Nigrosporins B Induced Apoptosis in Ca Ski Cells

**Figure 2:**
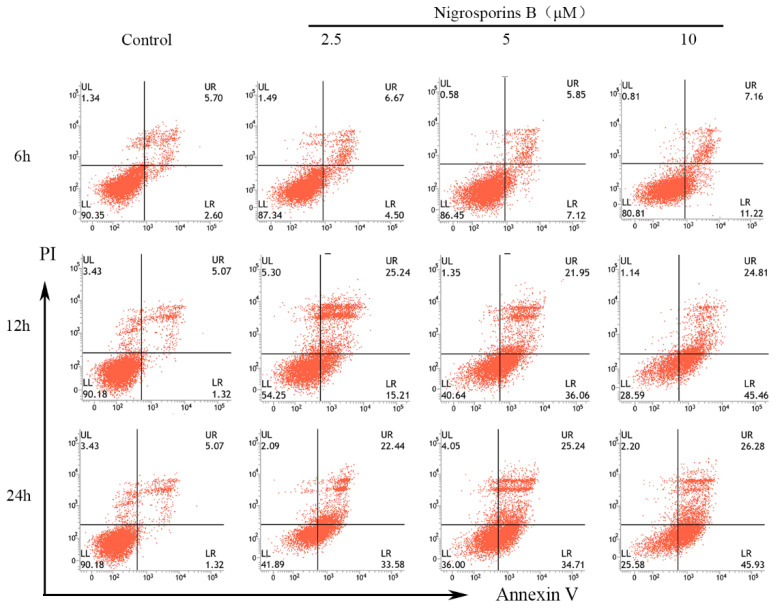
Nigrosporins B induces dose- and time-dependent apoptosis in Ca Ski cells, cells were treated with Nigrosporins B (0, 2.5, 5, 10 µM) for 6 h, 12 h, 24 h, then detected by flow cytometry. The horizontal coordinate-axis X is the value of Annexin V, the longitudinal coordinate-axis Y is the value of PI. Annexin V^−^/PI^−^ quadrant (LL) represents normal living cells, Annexin V^−^/PI^+^ quadrant (UL) represents necrotic cells, Annexin V^+^/PI^−^ quadrant (LR) represents early apoptotic cells, Annexin V^+^/PI^+^ quadrant (UR) represents late apoptotic cells.

**Figure 3:**
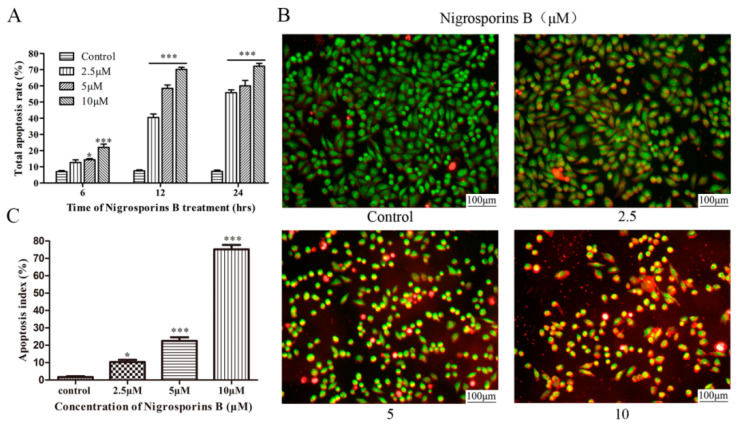
Nigrosporins B induced apoptosis in Ca Ski cells. (**A**) The total apoptotic cells proportion treated with Nigrosporins B (0, 2.5, 5, 10 µM) for 6 h, 12 h, 24 h were counted respectively; (**B**,**C**) AO/EB dual fluorescence staining to detect apoptosis in Ca Ski cells, cells treated with Nigrosporins B (0, 2.5, 5, 10 µM) for 24 h. Scale bar: 100 µM. Normal cells, showed green showed green fluorescence; early-stage apoptotic cells showed yellow-green fluorescence; late-stage apoptotic cells showed orange fluorescence; necrotic cells showed red fluorescence (* *p* < 0.05, *** *p* < 0.001).

To ascertain the mechanism of Ca Ski cells death acted by Nigrosporins B, apoptosis cells were identified by Annexin V-FITC/PI double staining assay. As shown in Figure 2, Ca Ski cells have appeared apoptosis after treated with Nigrosporins B of 10 μM for 6 h. The obvious alterations occurred at 12 h, there was a great increase of apoptotic cells proportion, especially for early apoptosis, were observed from 1.32% (control group) to 15.21%, 36.06% and 45.46% treated with Nigrosporins B of 2.5, 5 and 10 µM, respectively. The same trend was also observed after treatment for 24 h and the early apoptotic cells proportion increased to 33.58%, 34.71% and 45.93%. Meanwhile, the late apoptosis were also significantly increased under the same treatment conditions. The total apoptosis ratios were calculated as the sum of early apoptotic and late apoptotic cells and the results were shown in Figure 3A. These results suggested that Nigrosporins B could obvious induced Ca Ski cells apoptosis in a dose- and time-dependent manner.

We further using AO/EB dual fluorescence staining assays evaluated the apoptotic ratio in vitro, visualized under a fluorescent microscope. According to associated changes of cell membranes during the process of apoptosis, a clear distinction is made between normal cells, early and late apoptotic cells, and necrotic cells. Early-stage apoptotic cells were marked by crescent-shaped or granular yellow-green AO nuclear staining. Late-stage apoptotic cells were marked with concentrated and asymmetrically localized orange nuclear ethidium bromide staining. Necrotic cells increased in volume and showed uneven red fluorescence at their periphery. As the result showed (Figure 3B,C), after treated with Nigrosporins B of 2.5, 5 and 10 µM for 24 h, the number of normal cells (green) visibly decreased and the number of early apoptotic cells (yellow-green) and late apoptotic cells (orange) increased significantly.

### 3.3. Effect of Nigrosporins B on Mitochondrial Membrane Potential (MMP)

**Figure 4:**
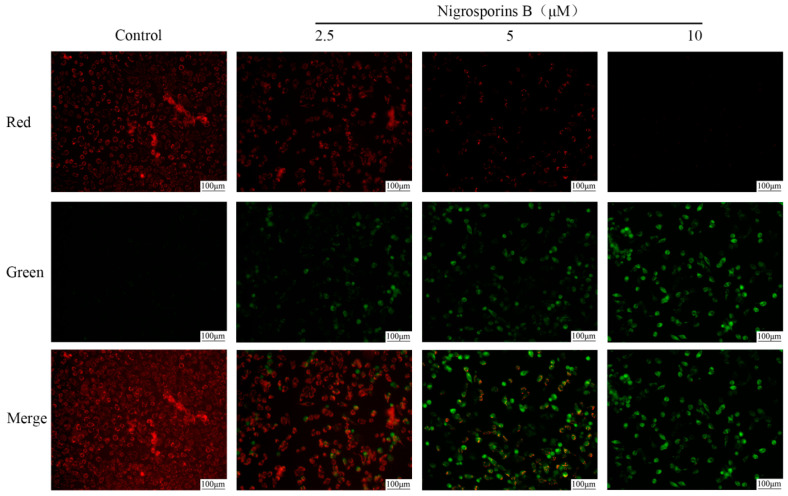
Using JC-1 fluorescence mitochondrial imaging assess the depolarization of MMP in Ca Ski cells, cells treated with Nigrosporins B (0, 2.5, 5, 10 µM) for 24 h. Scale bar: 100 µM. At high MMP, JC-1 exists as polymer emitted red fluorescence; at low MMP, JC-1 exists as a monomer and emitted green fluorescence.

To investigate the underlying mechanism of apoptosis induced by Nigrosporins B, JC-1 fluorescence probe was used to detect the alterations of MMP in Ca Ski cells with a fluorescence microscope. When the MMP was depolarized, JC-1 formed a monomer and emitted green fluorescence, and JC-1 aggregates emitted red fluorescence. As shown in Figure 4, treated with Nigrosporins B of 2.5, 5 and 10 µM for 24 h, the Ca Ski cells showed the gradual weakened red fluorescence and gradual enhanced green fluorescence compared to those in the control, which indicated the the damage of mitochondrial membrane and loss of ΔΨm. These results suggested that Nigrosporins B induced the depletion of the MMP, and this finding provided an insight into the mechanism of apoptosis in Ca Ski cells.

### 3.4. Effect of Nigrosporins B on Bcl-2 Family and Caspase Family Members

**Figure 5:**
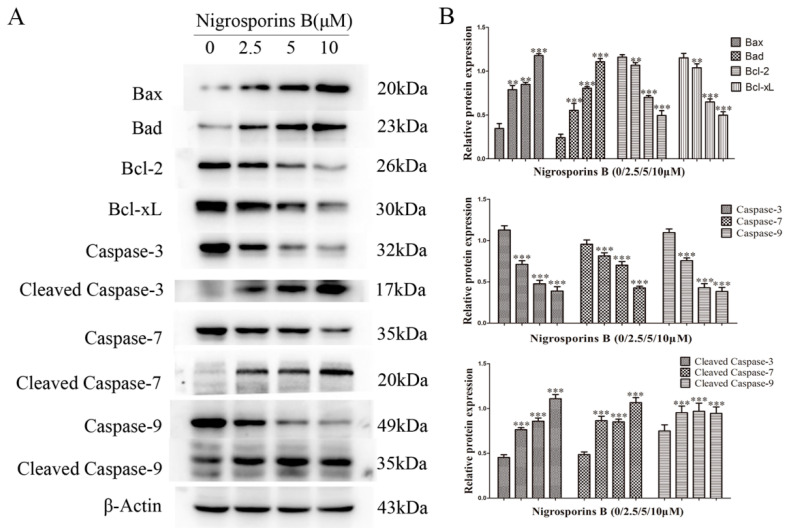
Nigrosporins B affected the expression of apoptosis-related proteins in Ca Ski cells, cells were treated with Nigrosporins B for 24 h. (**A**) Representative western blotting bands of Bad, Bax, Bcl-2, Bcl-xL, Caspase-3, -7 and -9 and Cleaved Caspase-3, -7 and -9 in Ca Ski cells; (**B**) Protein expression of Bad, Bax, Bcl-2, Bcl-xL, Caspase-3, -7 and -9 and Cleaved Caspase-3, -7 and -9 (** *p* < 0.01, *** *p* < 0.001).

The Bcl-2 family members are the crucial checkpoints for the mitochondrial-mediated apoptosis pathway [23]. As shown in Figure 5, Ca Ski cells were treated with Nigrosporins B of 2.5, 5 and 10 µM for 24 h, compared with the control group, the expression levels of the anti-apoptotic proteins Bcl-2 and Bcl-xL were remarkably reduced (*p* < 0.01 and *p* < 0.001), whereas the expression levels of pro-apotosis proteins Bax and Bad were significantly increased (*p* < 0.01 and *p* < 0.001). These results further hinted that the molecular mechanism of apoptosis might be via mitochondrial pathway.

In mitochondria pathway, pro-apoptotic proteins Bax and Bad translocate to mitochondria to mediate the release of cytochrome C into cytosol. This trigging the assembly of Caspase-9, which then activates Caspase-3-7, leads to cell death. Thus, Caspase-9/3/7 are specifically associated with the mitochondrial mediated apoptosis [24]. As shown in Figure 5, Ca Ski cells were treated with Nigrosporins B of 2.5, 5 and 10 µM for 24 h, compared with the control group, the expression levels of Caspase-9/3/7 were remarkably reduced (*p* < 0.001) and whereas the expression levels of Cleaved Caspase-9/3/7 were significantly increased (*p* < 0.001). The above results confirmed that Nigrosporins B induced apoptosis of Ca Ski cells involving the mitochondrial dependent intrinsic pathway.

### 3.5. Nigrosporins B Induced Autophagy in Ca Ski Cells

**Figure 6:**
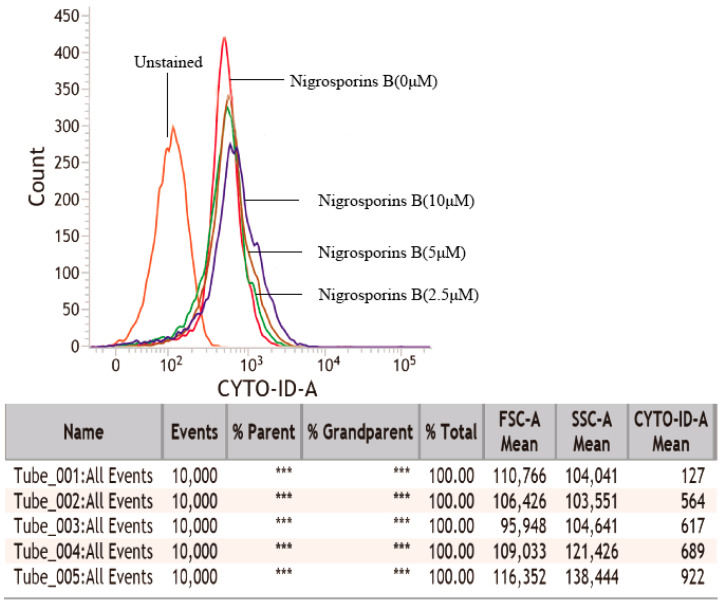
Cyto-ID staining to detect the relative number of autophagic vacuoles in Ca Ski cells. Cells were treated with Nigrosporins B (0, 2.5, 5, 10 µM) for 18 h. After staining with Cyto-ID Green Detection Reagent, analyzed by flow cytometry. Results are presented as histogram overlay. Tube_001 to Tube_005 in table represent the mean fluorescence intensity of unstained (background fluorescence intensity), normal cells and cells treated with Nigrosporins B (2.5, 5, 10 µM).(*** means not detected).

Previous studies [25] had highlighted the essential role of autophagic cell death induced by anti-cancer agents in the process of apoptosis. Therefore, we also explored whether Nigrosporins B could induce autophagy in this study. Considering that the formation of autophagic vacuoles are the identifying events of the initiation of autophagy process [26], we first using Cyto-ID autophagy detection kit to investigate the presence of autophagic vacuoles. As shown in Figure 6, Ca Ski cells were treated with Nigrosporins B of 2.5, 5 and 10 µM for 24 h, compared with the control group, the mean fluorescence intensity increased from 564 to 922, indicated that exposed to Nigrosporins B resulted in a possible autophagy induction in Ca Ski cells.

**Figure 7:**
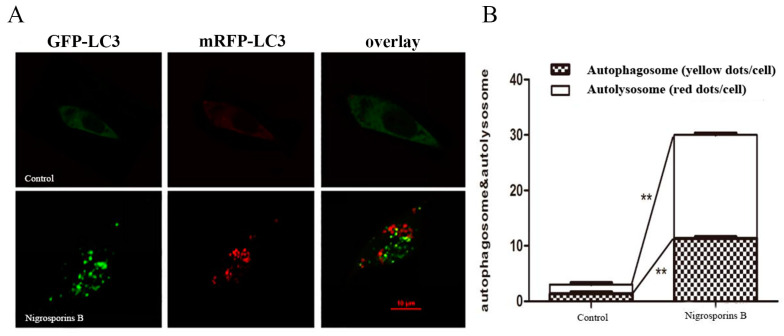
Nigrosporins B induced autophagic flux in Ca Ski cells. Ca Ski cells transfected with mRFP-GFP-LC3 were treated with Nigrosporins B for 18 h. (**A**) Cells transfected with mRFP-GFP-LC3 adenovirus were recorded by a confocal fluorescence microscopy; (**B**) The analysis of red dots and yellow dots in Ca Ski cells (** *p* < 0.01).

Several reports [27,28] had showed that the increase of autophagic vacuoles or autophagosomes cannot directly represent the true level of autophagy owing to autophagy was a highly dynamic, multi-step process, and take autophagic flux as the measurement standard of autophagy level has became the consensus of researchers at present. So, we subsequently examined the effect of Nigrosporins B on autophagic flux. A tandem mRFP-GFP-LC3 adenovirus was transfected into Ca Ski cells for 6.5 h and subsequently treated the cells with Nigrosporins B of 5 µM for 18 h. Obvious increases in the number of autophagosomes (yellow puncta) and autolysosomes (red-only puncta) were observed (Figure 7A). Meanwhile, the number of red-only dots increased more than yellow puncta when merged the two color together, these results suggested that Nigrosporins B treatment increased the autophagic flux significantly (Figure 7B).

### 3.6. Nigrosporins B Altered Expression of Autophagic Related Proteins

**Figure 8:**
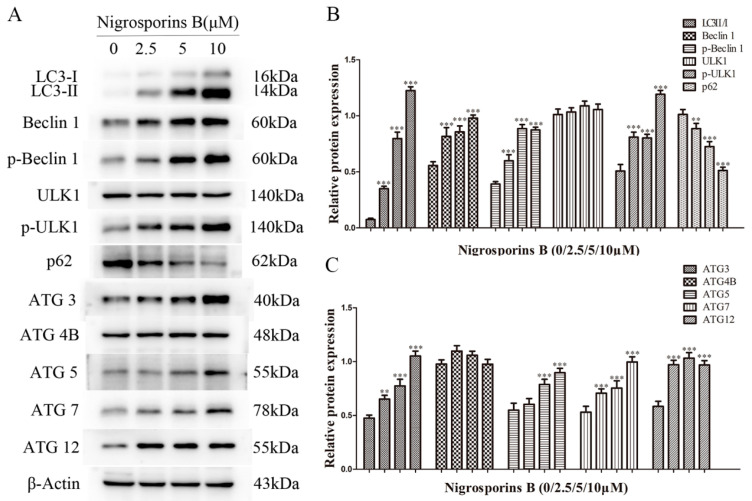
Nigrosporins B affected the expression of autophagy-related proteins in Ca Ski cells, cells were treated with Nigrosporins B for 24 h. (**A**) Representative western blotting bands of LC3-II/LC3-I, Beclin-1, p-Beclin-1, ULK1, p-ULK1, p62, ATG3, ATG4B, ATG5, ATG7 and ATG12; (**B**,**C**) Protein expression of LC3 II/I, Beclin-1, p-Beclin-1, ULK1, p-ULK1, p62, ATG3, ATG4B, ATG5, ATG7 and ATG12 (** *p* < 0.01, *** *p* < 0.001).

As a reliable marker of autophagy, the conversion of LC3-I into its autophagosome bound form LC3-II has been widely used for estimating the initiation of autophagy, simultaneously, some autophagy-specific proteins are also involved in the autophagy process as the key regulators, such as Beclin 1, p62, ULK1, ATG 3, ATG 4B, ATG 5, ATG 7 and ATG 12, et al. [26]. Therefore, we examined the expression levels of above proteins by western blot assay. The results (Figure 8A–C) revealed that Nigrosporins B treatment significantly up-regulated the ratio of LC3-II/I and increased the expression level of other autophagic related proteins, including Beclin 1, p-Beclin 1, p-ULK1, ATG 3, ATG 5, ATG 7 and ATG 12 in a dose-dependent manner. Whereas the expression levels of p62 protein were decreased and no significant differences for the proteins of ULK1 and ATG 4B compared with the control group. These results further confirmed that Nigrosporins B induced significant autophagy in Ca Ski cells.

### 3.7. Inhibition of Autophagy Increased the Cytotoxicity of Nigrosporins B

Accumulating evidence had demonstrated that autophagy played a “double-edged sword” role in cancer treatment as it could either promote or suppress the survival and proliferation of the tumor cells [8,29]. Thus, it is essential to further elucidate the properties of autophagy induced by Nigrosporins B in Ca Ski cells. We used three well-established small molecule autophagy inhibitors, namely 3-MA, CQ and Baf A1 combination with Nigrosporins B treated Ca Ski cells to investigate the mechanism of Nigrosporins B-induced autophagy. 3-MA is capable of interrupting the formation of autophagosome by inhibiting the expression of class Ⅲ PI3K protein [30], CQ is capable of blocking the fusion of autophagosomes and lysosomes by increasing the pH of lysosomes [31] and Baf A1 is capable of inhibiting autophagy by preventing the degradation of autophagolysosomes [32].

**Figure 9:**
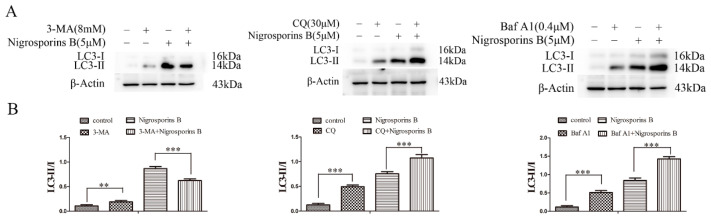
Nigrosporins B combined with autophagy inhibitors affected autophagy of Ca Ski cells, cells were treated with autophagy inhibitors for 6 h, then treated with Nigrosporins B for 24 h. (**A**) Nigrosporins B combined with or without 3-MA, CQ and Baf A1 affected the expression of LC3-II/LC3-I; (**B**) Protein expression of LC3 II/ I (** *p* < 0.01, *** *p* < 0.001).

**Figure 10:**
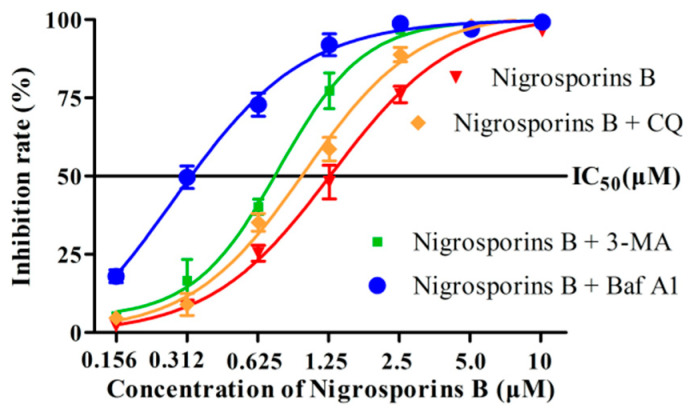
Nigrosporins B combined with or without 3-MA, CQ and Baf A1 inhibited the proliferation of Ca Ski cells, cells were treated with 3-MA, CQ and Baf A1 for 6 h, then treated with Nigrosporins B with different concentrations (10, 5, 2.5, 1.25, 0.625, 0.312, 0.156 µM) for 48 h, and cell viability was measured by MTT assay.

As shown in Figure 9A,B, when co-treatment with 3-MA (8 mM) and Nigrosporins B (5 μM), the ratio of LC3-II/LC3-I decreased compared with Nigrosporins B alone, indicated that 3-MA blocked the autophagic flux process of Ca ski cells induced by Nigrosporins B in the formation stage of autophagosomes (*p* < 0.001). When treated with CQ (30 μM) only, the ratio of LC3-II/LC3-I increased, indicated that CQ prevented the fusion of autophagosomes and lysosomes, which resulted in the interruption of autophagic flux process and the accumulation of autophagosomes in cells (*p* < 0.001). Co-treatment with CQ and Nigrosporins B led to further accumulation of autophagosomes, suggested that Nigrosporins B induced autophagy is a continual process. The effect of Baf A1 (0.4 μM) on the autophagic flux process of Ca ski cells induced by Nigrosporins B was similar to that of CQ (*p* < 0.001), but it blocked theautophagic flux process at the degradation stage of autophagolysosomes and the influence was stronger (*p* < 0.001). In addition, the the IC_50_ values respectively reduced from 1.24 μM to 0.68, 0.84 and 0.31 μM, when different concentrations of Nigrosporins B combined with 3-MA, CQ and Baf A1 (Figure 10). Thus, these results suggested that inhibition of autophagy could significantly enhanced the cytotoxic activity of Nigrosporins B and Nigrosporins B-induced autophagy has a protective effect against Ca Ski cells death. 

### 3.8. Nigrosporins B Inhibited the PI3K/AKT/mTOR Signaling Pathway in Ca Ski Cells

**Figure 11:**
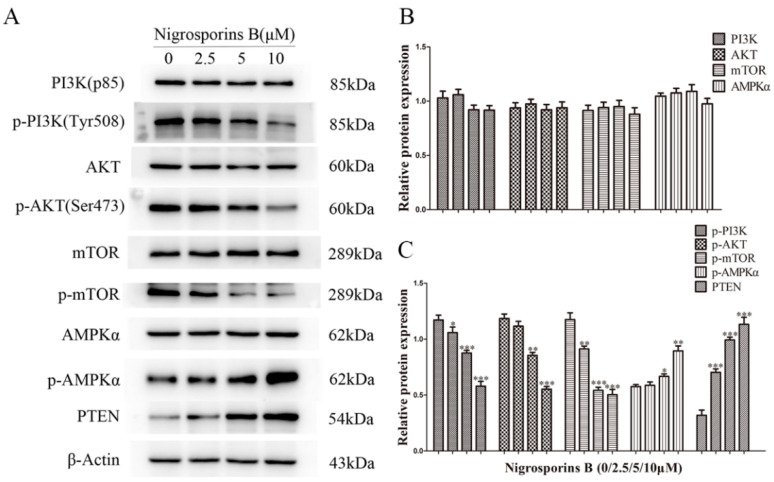
Nigrosporins B affected the expression of proteins of PI3K/AKT/mTOR signaling pathway in Ca Ski cells, cells were treated with Nigrosporins B for 24 h. (**A**) Representative western blotting bands of PI3K, Akt, mTOR, p-PI3K, p-Akt, p-mTOR, AMPKα, p-AMPKα and PTEN; (**B**,**C**) Protein expression of PI3K, Akt, mTOR, p-PI3K, p-Akt, p-mTOR, AMPKα, p-AMPKα and PTEN (* *p* < 0.05, ** *p* < 0.01, *** *p* < 0.001).

To investigate the role of PI3K/AKT/mTOR signaling pathway in Nigrosporins B-induced apoptosis and autophagy, the protein expression levels of up/down-stream key regulatory components were examined by western blot assay. As shown in Figure 11A–C, after treated with different concentrations of Nigrosporins B, the protein expression levels of p-PI3K, p-AKT and p-mTOR were decreased remarkably and the protein expression levels of p-AMPKα and PTEN levels were increased significantly. Whereas the expression of PI3K, mTOR, AKT and AMPKα remained constant. These results suggested that Nigrosporins B might induced apoptosis and autophagy through the suppression of the PI3K/AKT/mTOR pathway.

## 4. Discussion

Marine fungus secondary metabolites are one of the most copious sources of pharmacological active lead compounds currently, which have constantly provided a large number of valuable anti-cancer agents and a growing number of compounds are entering clinical trials or been marketed, such as Plinabulin, Trabectedi, Eribuli, et al. [18]. Nigrosporins B, a secondary metabolite of marine fungi, had also been obtained from the fungus *Nigrospora oryzae* which was originated from a lesion on a diseased fern [20], but its application in the pharmaceutical industry has not been researched. In this study, we reported the excellent anti-tumor activities of Nigrosporins B. Nigrosporins B exhibited stronger cytotoxicity in human cervical cancer Ca Ski cells compared to other tumor cells tested and fine selectivity which were demonstrated by the analysis of two types of normal cells. Taken together, these results suggested that Nigrosporins B acted as an anti-cancer agent in vitro, especially against cervical cancer.

Based on the above findings, the potential molecular mechanisms underlying its anti-cervical cancer effects were deeply clarified focus on the the process of PCD and related signaling pathway. Our results showed that Nigrosporins B might be a potent inducer of apoptosis in Ca Ski cells as evidenced by a dose-dependent increase number of apoptotic cells with FCM detection and AO/EB double fluorescence staining assay. Subsequently, Nigrosporins B treatment with different concentrations significantly enhanced the depolarization of MMP in Ca Ski cells and increased the expression levels of pro-apoptotic proteins Bax and Bad, decreased the expression levels of anti-apoptotic proteins Bcl-2 and Bcl-xL, these Bcl-2 family members are located on the outer mitochondrial membrane and governs mitochondrial membrane permeability. Following by, the activation of several key downstream caspases, which been identified by their cleaved status (autoproteolytic cleavage or cleavage other caspases at specific aspartic acid residues) [24], were analyzed by western blot assay. Research has shown that the expression levels of Cleaved Caspase-9, the most important initiator of mitochondrial pathway apoptosis, were obviously increased in the presence of Nigrosporins B stimulation and the same results were also shown in Cleaved Caspase-3 and 7 (the main effectors of apoptosis), which indicated the initiation and progression of caspase cascade that leads to apoptosis. These result suggested Nigrosporins B induced mitochondrial pathway mediated apoptosis in Ca Ski cells.

In addition, we also investigated autophagy in Ca Ski cells after treated with Nigrosporins B, and it turned out that Nigrosporins B induced obviously autophagy and significantly activated the autophagic flux in Ca Ski cells, which were demonstrated by measuring the formation of autophagic vacuoles and the number of autophagosomes and autolysosomes. Autophagy is a complex physiological process and numerous proteins and regulatory factors are involved in. In generally, autophagy begins with ULK1 that phosphorylates Beclin 1 protein which regulate autophagosome synthesis and autophagosome maturation [33]. During the formation of mammalian autophagosomes, ATG 12-conjugation and LC3-modification are required. ATG 12 is activated by ATG 7 and then conjugated to ATG 5 to form an autophagosomal precursor [34,35]. ATG 3 mediates LC3 lipidation also lead to the conversion of LC3-I into LC3-II, which has been shown is a mammalian autophagosome marker, and its level is correlated with the number of autophagosomes [36]. ATG 4B plays a central role in the LC3 lipid conjugation system, which is essential for the late step of autophagosome formation [37]. In addition, Beclin 1 is involved in the early steps of autophagic vesicle formation and P62 recruited to autophagosome with the increase of autophagy flux [33]. In this study, we evaluated the changes of some marker proteins (such as LC3-I/II, p62, Beclin 1 and ULK1) and important co-regulatory factors, especially ATG family members (such as ATG 3, 4B, 5, 7, 12), to further explored the molecular mechanism of Nigrosporins B induced autophagy. The result of western blot assay showed that the conversion of LC3-I into LC3-II and the expression of Beclin 1, p-Beclin 1, p-ULK1, ATG 3, 5, 7, 12 were all increased remarkably, whereas p62 was inhibited, which were consistent with the results of autophagy activation and autophagic flux processed.

Generally, it is thought that autophagy has opposing, context-dependent roles in cancer, including neutral, tumor-suppressive or tumor-promoting, which is determined by nutrient availability, microenvironment stress, pathogenic conditions, and the presence of immune system. Thus, there was an important question for the chemotherapy strategy of Nigrosporins B: Should we try to enhance autophagy or inhibit it? In this study, we tried to make a preliminary exploration by combining Nigrosporins B with three inhibitors which inhibit autophagy processes in different steps: 3-MA, CQ and Baf A1. The result of western blot assay showed that the application of three autophagy inhibitors all could block the process of autophagic flux induced by nigrosporins B, which was proved by the expression of LC3-II/I. Following autophagic flux was blocked, the sensitivity of Ca Ski cells to Nigrosporins B was significantly enhanced. Especially, Baf A1 owned the best synergistic effect to Nigrosporins B among them and the IC_50_ of Nigrosporins B decreased from 1.24 µM to 0.31 µM when combined with Baf A1. This may be owing to Baf A1 blocks the degradation of autophagolysosomes, the last checkpoint of autophagic flux, and the inhibition of autophagy process is the most thorough compared with other inhibitors. Additionally, Baf A1 could increase the ratio of Bax:Bcl-2 and cleaved CASP3 alone, thereby triggering an apoptotic response. Taken together, these findings suggested that Nigrosporins B induced protective autophagy, which might be a survival mechanism for Ca Ski cells to against the pressure of the external environment.

The PI3K/AKT/mTOR signaling pathway plays an important role in regulating the balance between cell proliferation, apoptosis and autophagy in response to cellular stress induced by chemotherapeutics in cancer cells. Abnormal PI3K/AKT/mTOR pathway are very common in human cancers and the activation or imbalance of this signaling pathway often favors tumor cells survival [38]. In this study, the protein expression and the phosphorylation were analyzed by western blot assay, the results showed that the intracellular content of protein PI3K, AKT, mTOR did not statistically changes in Ca Ski cells treated with Nigrosporins B, whereas their phosphorylation levels decreased obviously, which indicated that Nigrosporins B inhibited the PI3K/AKT/mTOR signaling pathway via inhibiting the phosphorylation of several key protein. Besides the above main components in the cascade response, PTEN and AMPKα, as known as the important tumor suppressors, deeply involved in the regulation of PI3K/AKT/mTOR signaling pathway as a negative regulator by inhibiting the phosphorylation of AKT and mTOR [39,40], respectively. The expression levels of PTEN and phosphorylated AMPKα protein all increased significantly in Ca Ski cells treated with Nigrosporins B and this further proved the suppression of Nigrosporins B on the PI3K/AKT/mTOR signaling pathway.

## 5. Conclusions

In this study, we demonstrated the effect of Nigrosporins B on the PCD pathways characterized by apoptosis and autophagy in Ca Ski cells for the first time. Nigrosporins B induced apoptosis of mitochondrial pathway and protective autophagy in Ca Ski cells which mediated by PI3K/AKT/mTOR signaling pathway. In addition, synergistic with autophagy inhibitor Baf A1 could enhanced the cytotoxicity of Nigrosporins B against Ca Ski cells significantly. FGFR pathway is a key upstream pathway of PI3K/AKT/mTOR pathway, and abnormal FGFR pathway are very common in cervical cancers [41], which give us a new sight for our next mechanism research. These findings will be helpful for the future applications of Nigrosporins B as a cervical cancer therapeutic agent.

## Data Availability

The datasets used and analyzed during the current study are available from the corresponding author on reasonable request.
